# Author Correction: nc886 is induced by TGF-β and suppresses the microRNA pathway in ovarian cancer

**DOI:** 10.1038/s41467-018-07818-2

**Published:** 2018-12-19

**Authors:** Ji-Hye Ahn, Hyun-Sung Lee, Ju-Seog Lee, Yeon-Su Lee, Jong-Lyul Park, Seon-Young Kim, Jung-Ah Hwang, Nawapol Kunkeaw, Sung Yun Jung, Tae Jin Kim, Kwang-Soo Lee, Sung Ho Jeon, Inhan Lee, Betty H. Johnson, Jung-Hye Choi, Yong Sun Lee

**Affiliations:** 1 Department of Life and Nanopharmaceutical Sciences and Department of Oriental Pharmaceutical Science, Kyung Hee University, Seoul, 02447 Korea; 2 Division of Thoracic Surgery, Michael E. DeBakey Department of Surgery, Baylor College of Medicine, Houston, TX 77030 USA; 3 Department of Systems Biology, University of Texas MD Anderson Cancer Center, Houston, TX 77030 USA; 4 Rare Cancer Branch, Research Institute, National Cancer Center, Goyang, 10408 Korea; 5 Medical Genomics Research Center, KRIBB, Daejeon, 34141 Korea; 6 Genomics Core Laboratory, Omics Core Laboratory, Research Institute, National Cancer Center, Goyang, 10408 Korea; 7 Department of Biochemistry and Molecular Biology, University of Texas Medical Branch, Galveston, TX 77555-1072 USA; 8 Institute of Molecular Biosciences, Mahidol University, Nakhon Pathom, 73170 Thailand; 9 Verna and Marrs McLean Department of Biochemistry and Molecular Biology, Baylor College of Medicine, Houston, TX 77030 USA; 10 Department of Obstetrics and Gynecology, Cheil General Hospital and Women’s Healthcare Center, College of Medicine Dankook University, Seoul, 04619 Korea; 11 Department of Life Science and Center for Aging and Health Care, Hallym University, Chuncheon, 24252 Korea; 12 miRcore, Ann Arbor, MI 48105 USA; 13 Department of Cancer Biomedical Science, Graduate School of Cancer Science and Policy, National Cancer Center, Goyang, 10408 Korea

Correction to: *Nat. Commun.*; 10.1038/s41467-018-03556-7; published online 21 March 2018.

In the original version of the Supplementary Information file associated with this Article, Supplementary Fig. 18 panel b was inadvertently replaced with a duplicate of panel a.

The error has now been fixed and the corrected version of the Supplementary Information PDF is available to download from the HTML version of the Article.

