# Supplementary material for: nc886 is induced by TGF-β and suppresses the microRNA pathway in ovarian cancer
Source: Nat Commun. 2018 Mar 21;9:1166. doi: 10.1038/s41467-018-03556-7 (PMC5862949; doi:10.1038/s41467-018-03556-7)
Supplement: Supplementary file 1 — Supplementary Information [file 41467_2018_3556_MOESM1_ESM.pdf]

Supplementary Information

"nc886 is induced by TGF-β and suppresses the microRNA pathway in ovarian cancer"

Ahn, et al

Supplementary Methods

Methylation specific-high resolution melting (MS-HRM)

Bisulfite treatment and PCR reactions for MS-HRM were done as described in the main text for EpiTPYER assays. PCR primers are listed in Supplementary Data File 9 and amplicons are shown in Supplementary Fig. 3a. Melting curves of MS-HRM were obtained in LightCycler® 480 Instrument II and were analyzed using GeneScanning software (Roche, Basel, Switzerland).

Chromatin immunoprecipitation (ChIP) assay

ChIP was performed using a ChIP Assay Kit (EMD Millipore, Billerica, MA), according to the manufacturer's protocol. Briefly, formaldehyde (final concentration of 1%)-fixed cells were harvested and lysed. Chromatin was sonicated to yield DNA fragments of 300-500 nucleotides (nt) and then immunoprecipitated with anti-SMAD4 antibody (sc-73599 from Santa Cruz Biotechnology, Dallas, TX) overnight at 4 ℃. The antibody-chromatin complex was pulled down with salmon sperm DNA-protein G-agarose beads. After reversing the cross-link, DNA (immunoprecipitated and input) was purified using a Fragment DNA purification kit (Intron Biotechnology, Seoul, Korea) and then PCR-amplified by using primers in Supplementary Data File 9.

Orthotopic implantation

BALB/c athymic nude mice (Orient Bio, Seongnam, Korea) were used for this study. All mice were females weighing 20–25 g and were housed under conventional laboratory conditions with chow and water available *ad libitum*. Metastasis of ovarian cells (“SKOV3_vector” and ‘SKOV3_nc886”) was induced by inoculating 5×10^6 cells (in 100 μl of PBS) into the peritoneal cavity of each mouse (total 20 mice; n=10 for each cell line). From the 5th week after inoculation and onwards, 1/2 of the mice were treated with TGF-β (1 μg kg^{-1} body weight) and the other 1/2 with the vehicle PBS via intraperitoneal injection. The treatment was 3× weekly; Monday, Wednesday, and Friday. At 8th week post-inoculation, mice were sacrificed and dissected. The number and location of individual tumor nodules were identified and recorded. The experimental protocol [KHUASP(SE)-16-088] was approved by the Institutional Animal Care and Use Committee of Kyung Hee University.

Luciferase assays

Luciferase assays were done by using a Dual-Luciferase® Reporter Assay System (Promega, Madison, WI). OSE80PC cells (in a 96-well plate) were transfected with 0.5 ng of indicated sensor plasmids (Supplementary Fig. 17b and Supplementary Table 4) and 5 ng of pRL-SV40 combined with miRNA mimic or control mimic RNA per well. At 24 hrs post-transfection, cells were harvested for luciferase assays according to the manufacturer’s instruction. Luciferase values were normalized and plotted as described in [1].

Mass spectrophotometry

Following streptavidin pull-down and subsequent wash steps, the eluted fractions were boiled in 30 µl of 1× NUPAGE® LDS sample buffer (Invitrogen, Carlsbad, CA) and subjected to SDS-PAGE (NuPAGE 4-12% Bis-Tris Gel, Invitrogen). Gel was visualized with Coomassie Brilliant Blue-stain and excised into 3 gel pieces according to the molecular size. The individual gel pieces were destained and subject to in-gel digestion using trypsin (T9600; GenDepot, Barker, TX). The tryptic peptide was resuspended in 10 µl of loading solution (5% methanol containing 0.1% formic acid) and subjected to nanoflow LC-MS/MS analysis with a nLC 1200 HPLC (Thermo Fisher Scientific, Waltham, MA) coupled to Orbitrap Fusion™ Lumos mass spectrometer (Thermo Fisher Scientific). The peptides were loaded onto a Reprosil-Pur Basic C18 (1.9 µm, Dr. Maisch GmbH, Germany) pre-column of 2 cm × 100 µm size. The pre-column was switched in-line with an in-housed 50 mm × 150 µm analytical column packed with Reprosil-Pur Basic C18 equilibrated in 0.1% formic acid in water. The peptides were eluted using a 35 min discontinuous gradient of 5-28% acetonitrile in 0.1% formic acid at a flow rate of 850 nl min^{-1}. The eluted peptides were directly electro-sprayed into mass spectrometer operated in the data-dependent acquisition mode acquiring fragmentation spectra of the top 30 strongest ions under direct control of Xcalibur software (Thermo Fisher Scientific). Parent MS spectrum was acquired in the Orbitrap with full MS range of 300-1400 m/z in the resolution of 120,000. HCD fragmented MS/MS spectrum was acquired in ion-trap with rapid scan mode. Obtained MS/MS spectra were searched against target-decoy Human refseq database (release June 2015, containing 73637 entries) in Proteome Discoverer 1.4 interface (Thermo Fisher Scientific) with Mascot algorithm (Mascot 2.4, Matrix Science). Variable modification of Oxidation of methionine was allowed. The precursor mass tolerance was confined within 20 ppm with fragment mass tolerance of 0.5 Dalton and a maximum of two missed cleavages was allowed. Assigned peptides were filtered with 1% false discovery rate (FDR) and subject to manual verifications. The intensity-based absolute quantification (iBAQ) algorithm was used to calculate protein abundance using an in-house data processing algorithm [2]. Simply, iBAQ was calculated based on normalization of summed peptide intensity divided by the number of theoretically observable tryptic peptide of certain protein. The proteins exclusively show iBAQ in nc886 pulled down sample were identified as nc886-specific binding proteins (listed in Supplementary Data File 7).

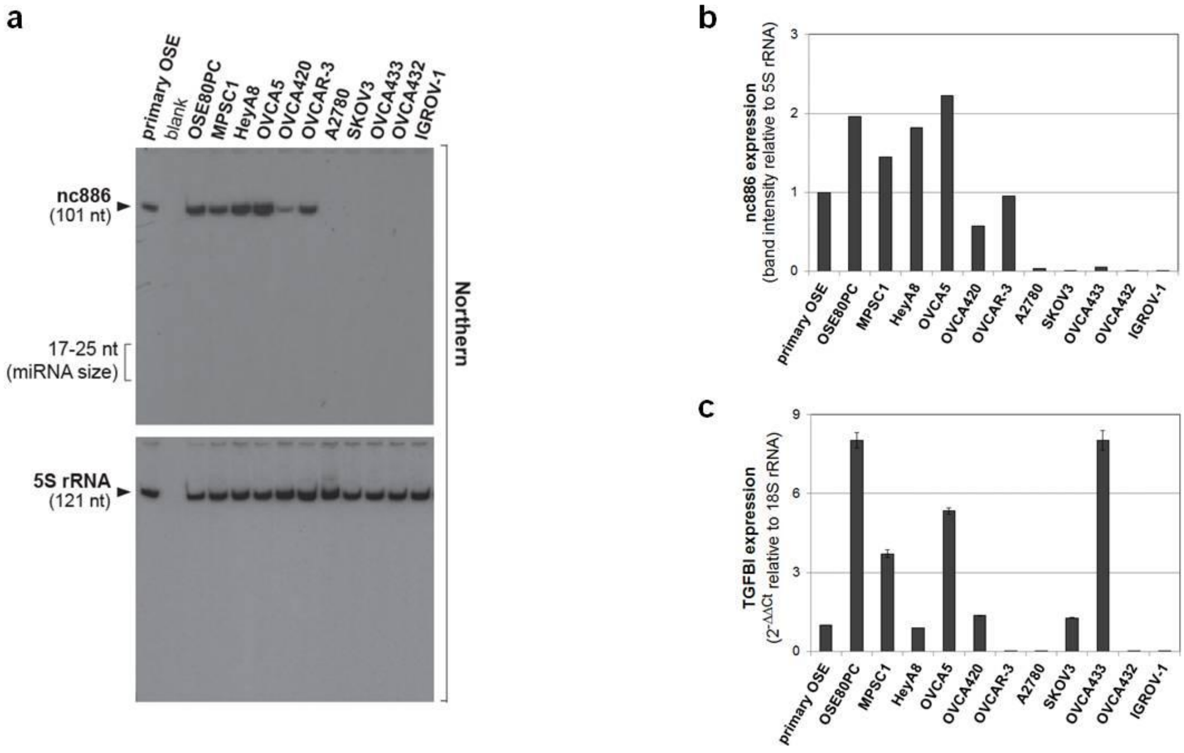

Supplementary Figure 1. nc886 and *TGFBI* expression in a panel of ovarian cell lines
a-b. Northern hybridization of nc886, together with 5S rRNA for equal loading in ovarian cell lines. Gel images are panel a and quantitation results are in panel b. Although a molecular size marker was not included in this gel, we could estimate the size of mature miRNA (17-25 nt), because we used a standard procedure for Northern hybridization and ensured the integrity of RNA resolution by checking the ethidium bromide (EtBr) staining of the gel. This is well shown in Supplementary Fig. 2.
c. qRT-PCR measurement of *TGFBI*. The y-axis is $2^{-\Delta\Delta Ct}$ values normalized to 18S rRNA and relative to primary OSE (that was set as 1). An average and a standard deviation were calculated from triplicate measurements and are shown in the graph. All qRT-PCR primers are summarized in Supplementary Data File 9.

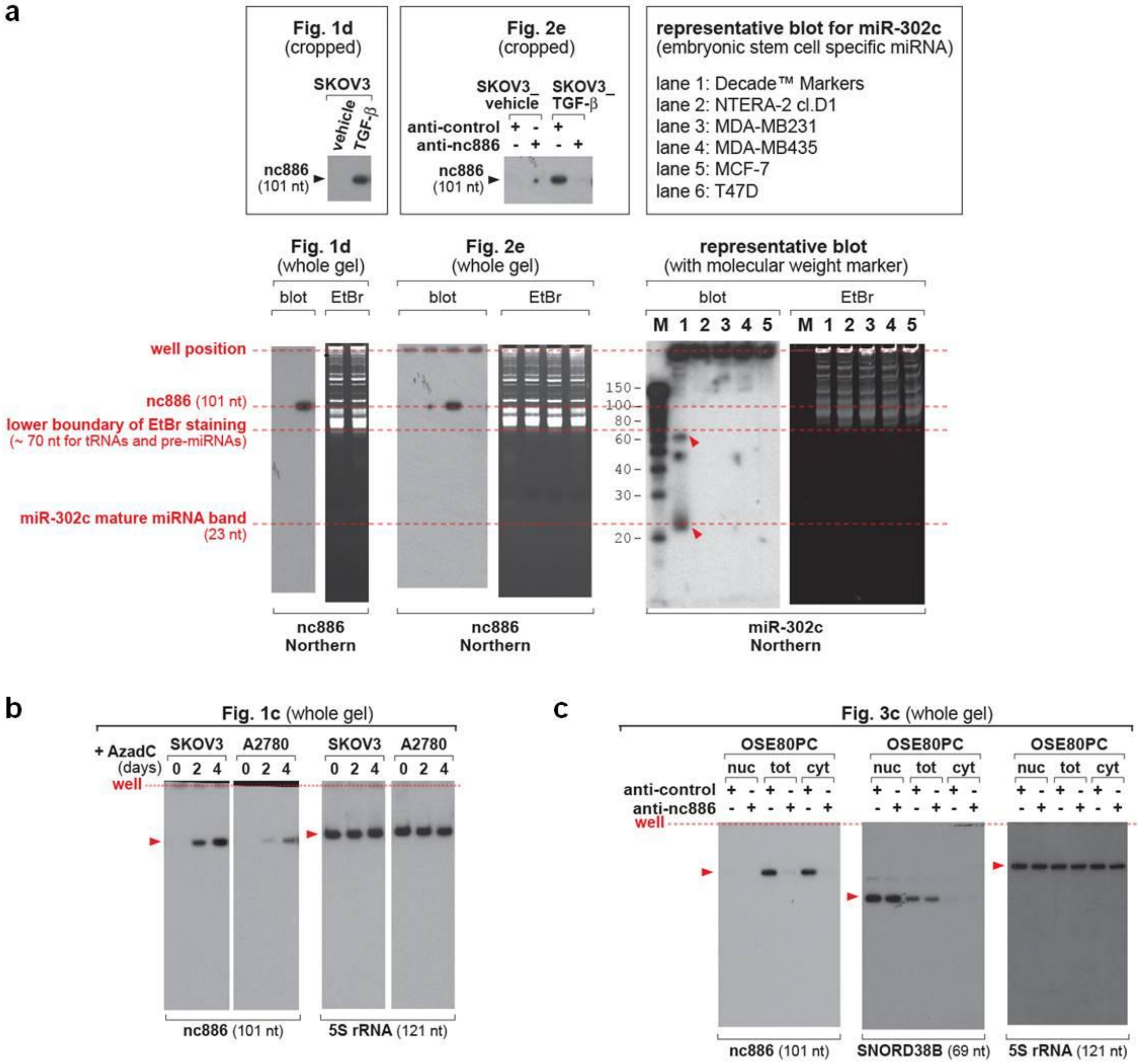
a
Fig. 1d
(cropped)
SKOV3
vehicle
TGF-β
nc886
(101 nt)
Fig. 2e
(cropped)
SKOV3_
vehicle
SKOV3_
TGF-β
anti-control
anti-nc886
nc886
(101 nt)
representative blot for miR-302c
(embryonic stem cell specific miRNA)
lane 1: Decade™ Markers
lane 2: NTERA-2 cl.D1
lane 3: MDA-MB231
lane 4: MDA-MB435
lane 5: MCF-7
lane 6: T47D
Fig. 1d
(whole gel)
Fig. 2e
(whole gel)
representative blot
(with molecular weight marker)
blot
EtBr
M 1 2 3 4 5
well position
nc886 (101 nt)
lower boundary of EtBr staining
(~ 70 nt for tRNAs and pre-miRNAs)
miR-302c mature miRNA band
(23 nt)
150
100
80
60
40
30
20
nc886
Northern
miR-302c
Northern
b
Fig. 1c (whole gel)
+ AzadC
(days)
SKOV3
A2780
0 2 4
well
nc886 (101 nt)
5S rRNA (121 nt)
c
Fig. 3c (whole gel)
OSE80PC
nuc
tot
cyt
anti-control
anti-nc886
well
nc886 (101 nt)
SNORD38B (69 nt)
5S rRNA (121 nt)

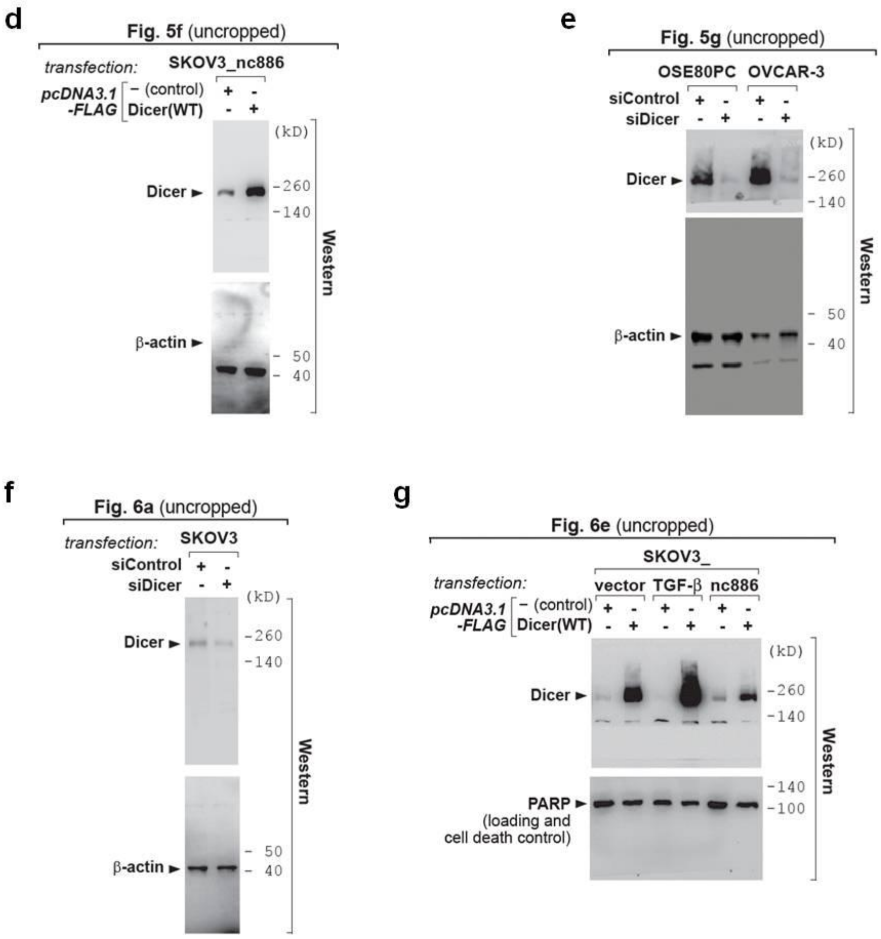

Supplementary Figure 2. Integrity of cropped images of our experiments

a. In our study, Northern hybridization of nc886 is one of the key data. Due to limited space, we cropped the autoradiogram images in all the main figures. Northern hybridization is a routine experiment in our laboratory and we follow a standard procedure; for example, the same 15% gel, electrophoresis for 100 min, *etc*. Because of this standardization, we could estimate the size of bands without any molecular size marker and so we did not include it. Instead, we checked all gels by examining the EtBr staining and made sure that the staining patterns were not abnormal. Here we show the autoradiogram of whole blots and the EtBr staining of whole gels for Fig. 1d and 2e. For comparison, we display in parallel a representative blot (one of our early blots which was done in 2009 when I set up Northern hybridization in my UTMB laboratory) containing Decade™ Markers (Ambion, Carlsbad, CA). EtBr staining patterns are well aligned and also individual bands are located at correct positions (nc886 at 101 nt, pre-miR-302c at ~60 nt, mature miR-302c at 23 nt). The early blot for comparison was intended to measure miR-302c, an embryonic stem cell specific miRNA. Mature miR-302c and its precursor (designated by red arrowheads) were detected only in an embryonic carcinoma cell line, NTERA-2 cl.D1. All our data proves the integrity of our Northern blots and the size estimation, without a molecular size marker.

b-g. Uncropped images of our Northern and Western blots.

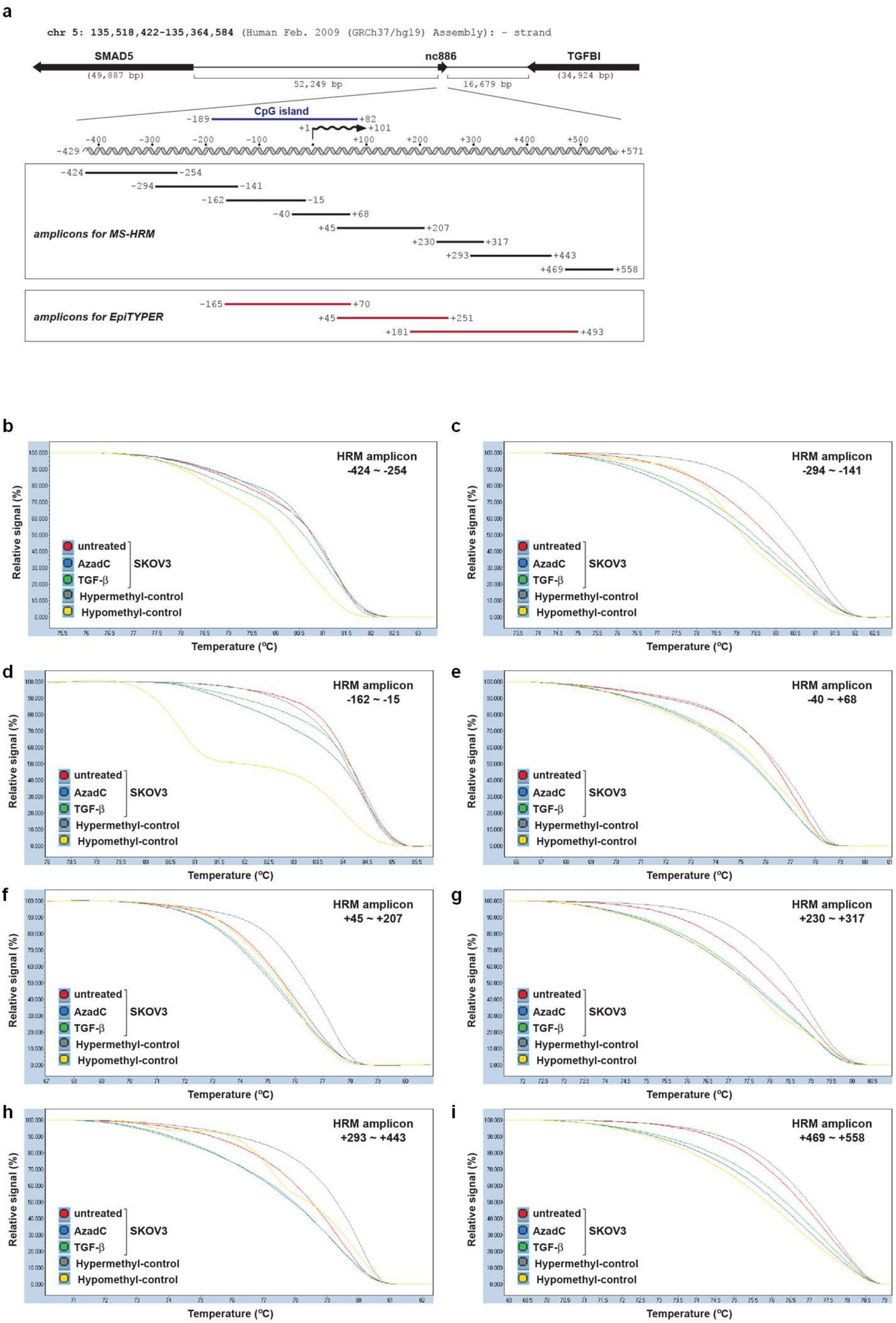
a
chr 5: 135,518,422-135,364,584 (Human Feb. 2009 (GRCh37/hg19) Assembly): - strand
SMAD5
nc886
TGFBI
(49,887 bp)
52,249 bp
16,679 bp
(34,924 bp)
CpG island
-189
+82
+1
+101
-429
+571
amplicons for MS-HRM
-424 -254
-294 -141
-162 -15
-40 +68
+45 +207
+230 +317
+293 +443
+469 +558
amplicons for EpiTYPER
-165 +70
+45 +251
+181 +493
b
HRM amplicon -424 ~ -254
c
HRM amplicon -294 ~ -141
d
HRM amplicon -162 ~ -15
e
HRM amplicon -40 ~ +68
f
HRM amplicon +45 ~ +207
g
HRM amplicon +230 ~ +317
h
HRM amplicon +293 ~ +443
i
HRM amplicon +469 ~ +558
untreated
AzadC
TGF-β
SKOV3
Hypermethyl-control
Hypomethyl-control
Relative signal (%)
Temperature (ºC)

Supplementary Figure 3. Hypomethylation of the nc886 genomic region by TGF-β

a. A diagram of the nc886 genomic region, which was modified from Fig. 1b. A wide nc886 locus spanning its flanking genes is shown on the top and a 1000 nt region is shown magnified on the bottom. The arrows indicate transcriptional direction. All symbols (nc886 RNA, wavy line; CpG island, blue bar; MS-HRM amplicons, black bars; EpiTYPER amplicons, dark magenta bars) on the magnified view are drawn to an exact scale, with their nt coordinates numbered according to the 5'-end of nc886 being +1.

b-i. Melting curves of indicated MS-HRM amplicons. SKOV3 cells were untreated or treated with 10 μM of AzadC or 10 ng ml^{-1} of TGF-β for 96 hrs. "Hypermethyl-control" and "Hypomethyl-control" are genomic DNA from 184B5ME and 184AA2 cell lines respectively. These 2 cell lines are human mammary epithelial cell lines obtained from Dr. Martha R. Stampfer at Lawrence Berkeley National Laboratory and have been shown to have 100% and 50% methylation at the nc886 genomic region [3]. Except for the amplicon -424~-254, all others showed that "AzadC" and "TGF-β" shifted melting curves toward that of 184AA2 ("Hypomethyl-control") as compared to "untreated". Therefore, we decided to perform EpiTYPER assays in DNA segments at -165 and downstream (EpiTYPER amplicons shown in panel a, see also Fig. 1b). In agreement with the MS-HRM data, EpiTYPER (and also pyrosequencing) proved that TGF-β decreased CpG methylation like AzadC (Fig. 1g-h).

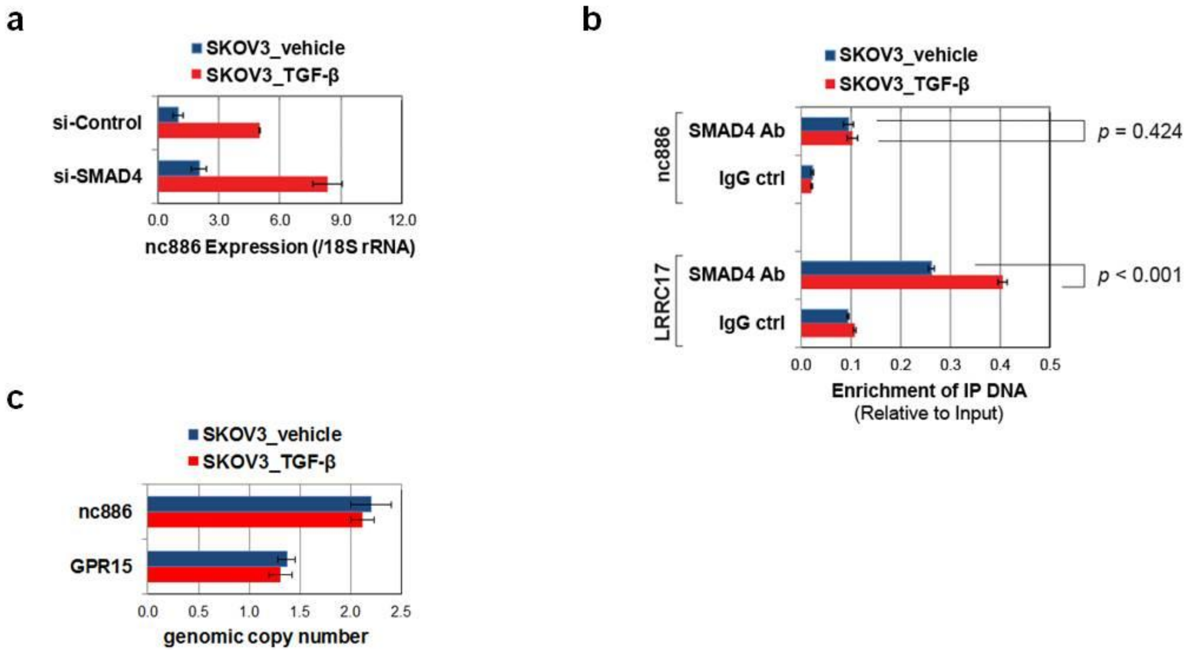

Supplementary Figure 4. nc886 induction by TGF-β is neither via the canonical SMAD pathway nor due to genomic amplification

a. qRT-PCR of nc886. siRNA against SMAD4 (catalog # 1141302 and #1141305) and control siRNA (catalog # SN-1003) were purchased from Bioneer (Daejeon, Korea). The 2 siRNAs targeting different sites of SMAD4 were used as an equimolar mixture. siRNAs at 40 nM were transfected at 48 hrs post-treatment of TGF-β at 10 ng ml^{-1}. Cells were harvested at 48 hrs afterwards. If nc886 induction by TGF-β was via the canonical SMAD pathway, si-SMAD4 would have negated that induction but our data indicated that was not the case.

b. ChIP assays to measure SMAD4 binding to nc886 or LRRC17 promoter regions. After TGF-β treatment for 96 hrs, ChIP assays were performed. After qPCR, the amounts of immunoprecipitated DNA relative to input DNA were calculated from $2^{-\Delta Ct}$ values. An average and the standard deviation from quadruplicate samples are shown. TGF-β did not increase SMAD4 binding to the nc886 region but did so to LPRC17, a previously known SMAD4 target gene [4]. Our ChIP data were in agreement with the absence of a SMAD4 binding site in the nc886 region in the previous SMAD4 ChIP-seq data [4] as well as in the *in silico* prediction by PROMO 3.0 (http://alggen.lsi.upc.edu/recerca/menu_recerca.html).

c. Real time-PCR measurement of nc886 on genomic DNA isolated from SKOV3 cells treated with vehicle or TGF-β for 96 hr. Ct values from SKOV3 cells were normalized to those of normal primary human mammary cells (184D cell line provided by Dr. Martha R. Stampfer [3]) and then converted to $2^{-\Delta\Delta Ct}$ values (x-axis). An average and a standard deviation are shown from triplicate samples. GPR15 was included as a reference locus. nc886 was amplified with “miR-886-5p sense” and “T7 pre-886 as”; GPR15 was with “GPR15 (F)” and “GPR15 (R)”. All primer sequences are shown in Supplementary Data File 9. The genomic copy numbers of these 2 genes were slightly different, presumably due to technical reasons such as PCR efficiency. Importantly, neither was induced by TGF-β and thus we assured that the induction was not due to a change in the genomic copy number.

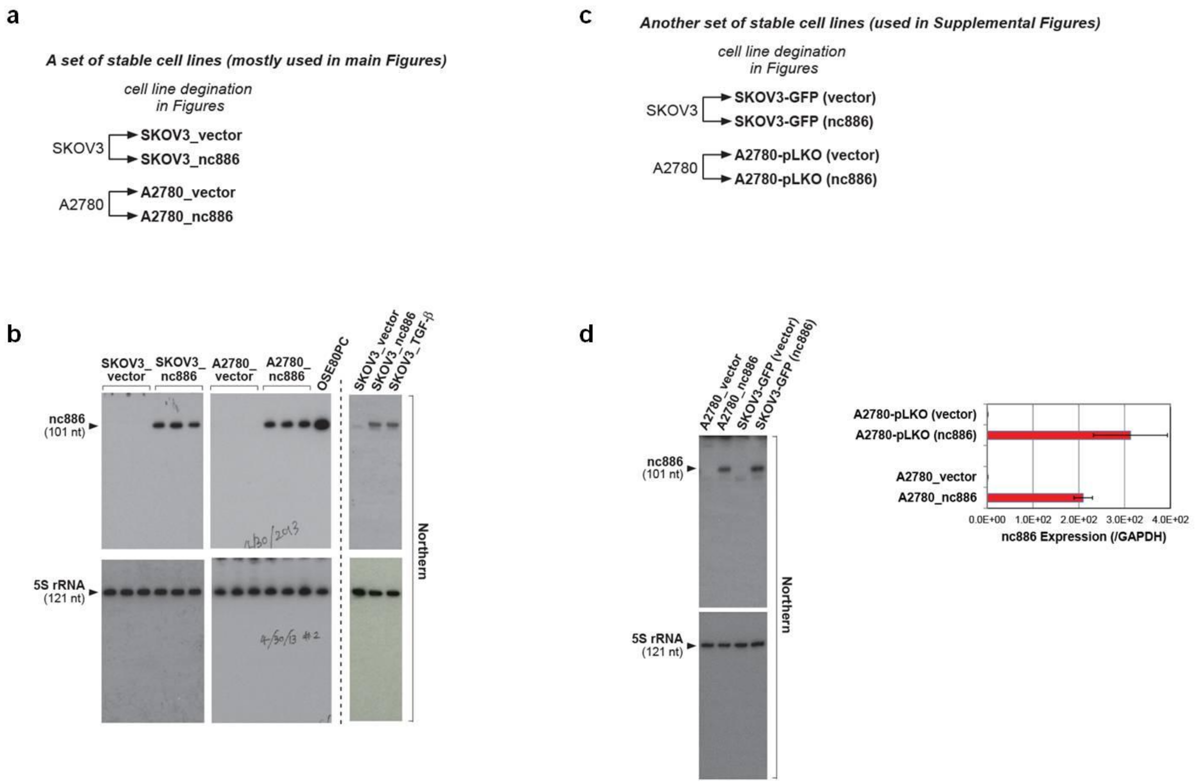

Supplementary Figure 5. OC lines stably expressing nc886

Nomenclature and validation of nc886-expressing OC lines derived from SKOV3 and A2780. The nomenclature in panel a and c is used consistently in all figures and tables and also in the text throughout this paper. nc886 expression was validated by Northern hybridization and qRT-PCR, and is shown in panel b and d. The panel d qRT-PCR was performed in the same way as in Supplementary Fig. 1c, except that GAPDH was used for normalization. Since Northern hybridization and qRT-PCR in each panel were done in the same batch of experiments, nc886 expression levels of the 4 stable OC lines were assured to be comparable among one another, similar to TGF-β treatment, and not higher than OSE80PC.

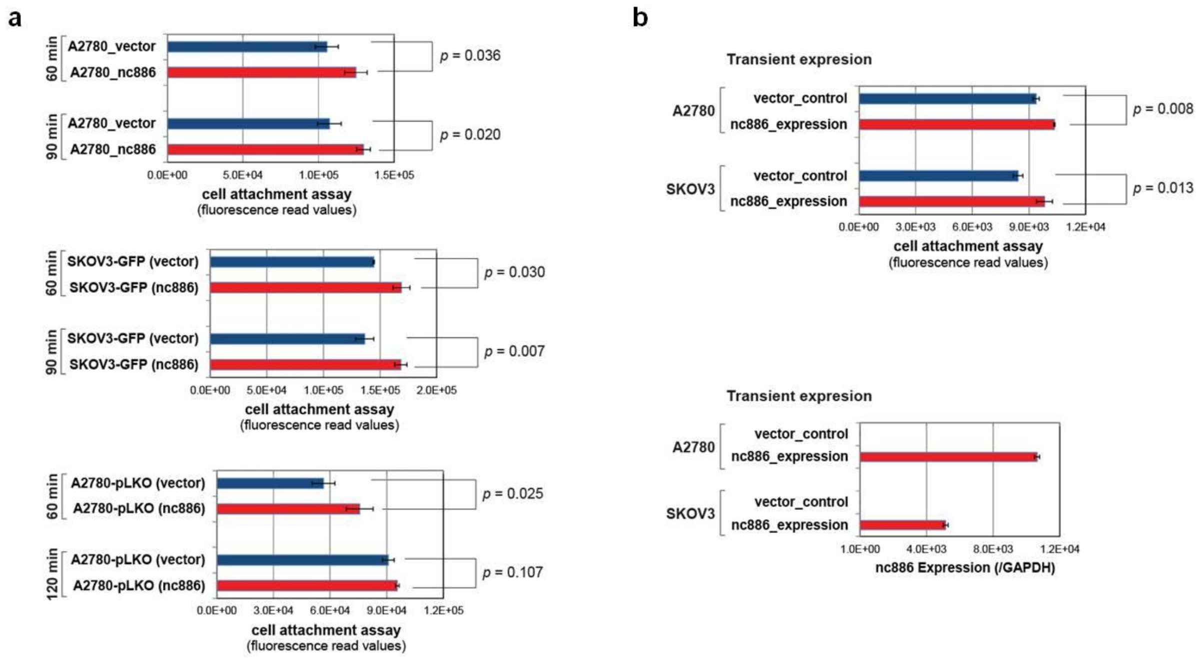

Supplementary Figure 6. Cell attachment assays

Cell attachment assays in stably nc886-expressing OC lines (panel a) and transiently nc886-expressing OC cells (panel b). In transient expression, assays were performed at 24 hrs post-transfection with the nc886-expressing plasmid or a control vector (see Supplementary Table 4 for plasmid information). For qRT-PCR measurement (panel b, bottom graph), cells were harvested after 24 hrs upon transfection.

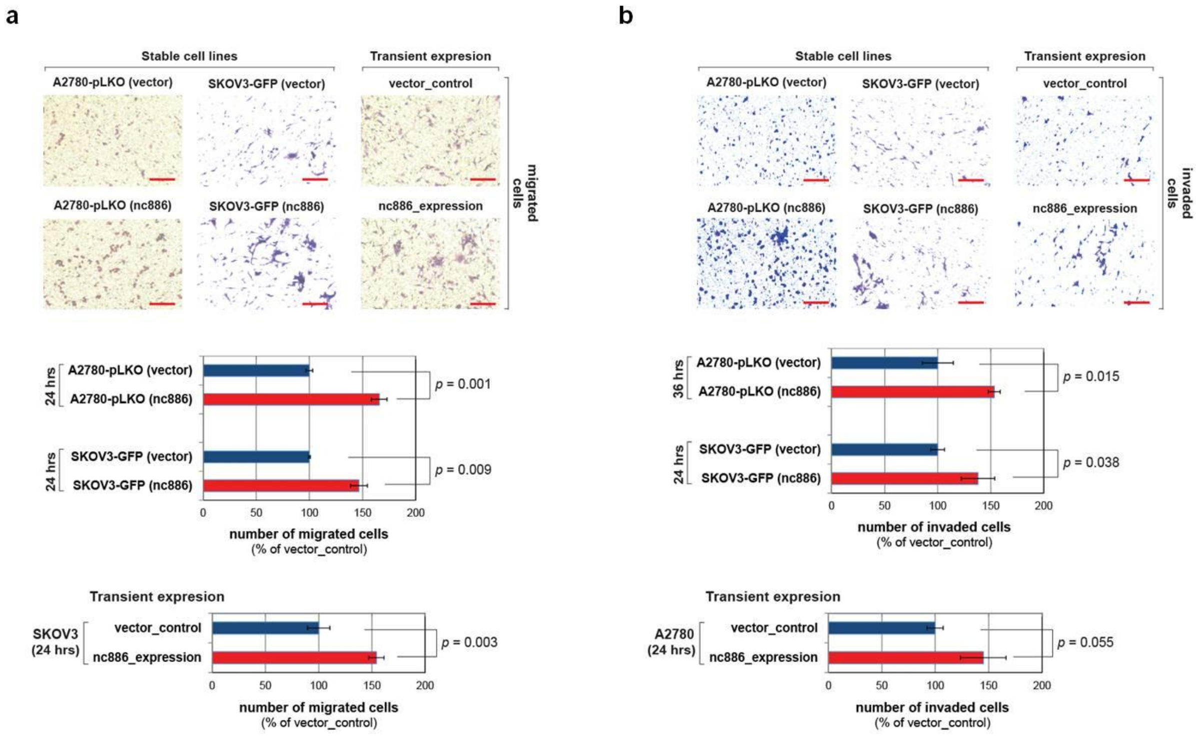

Supplementary Figure 7. Cell migration and invasion assays

Cell migration (panel a) and invasion assays (panel b) in indicated OC cells. In each panel, representative images (top) and quantification graphs (bottom) are displayed. In the graphs, an average and the standard deviation from pentaplicates are shown. Thin bars indicate 100 μm.

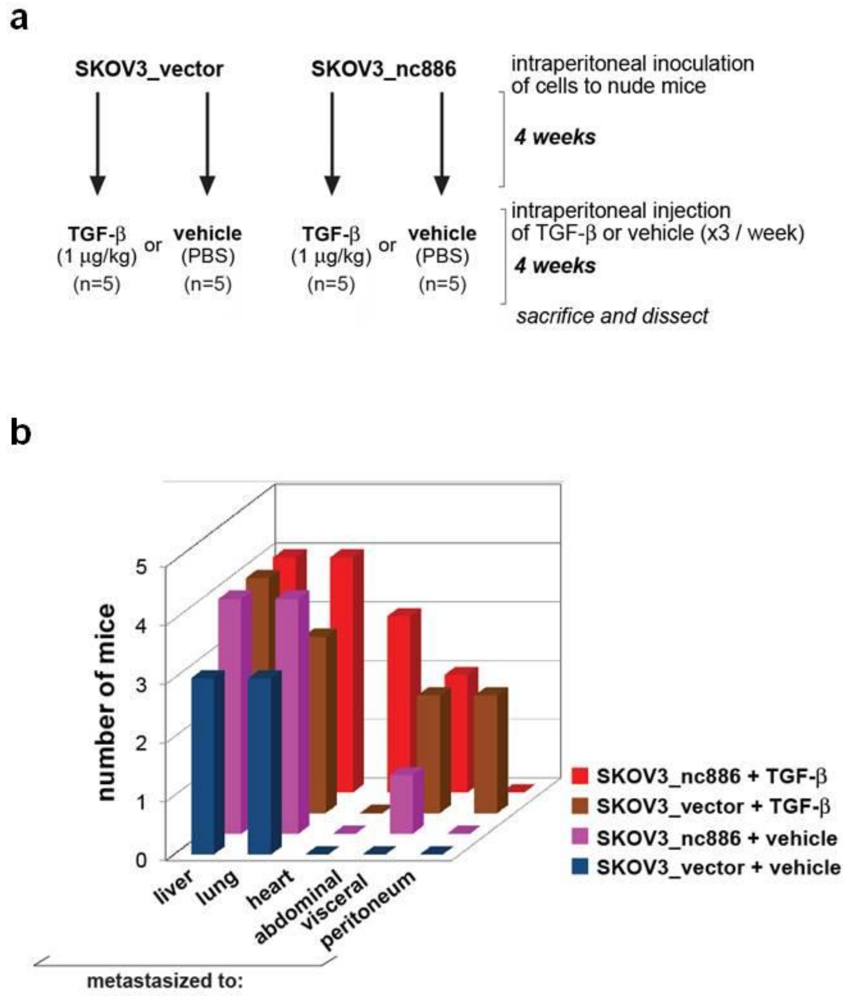

Supplementary Figure 8. Mouse experiment

a. An experimental scheme for orthotopic implantation of OC cells into nude mice.

b. The number of mice having OC cell metastasis to indicated organs.

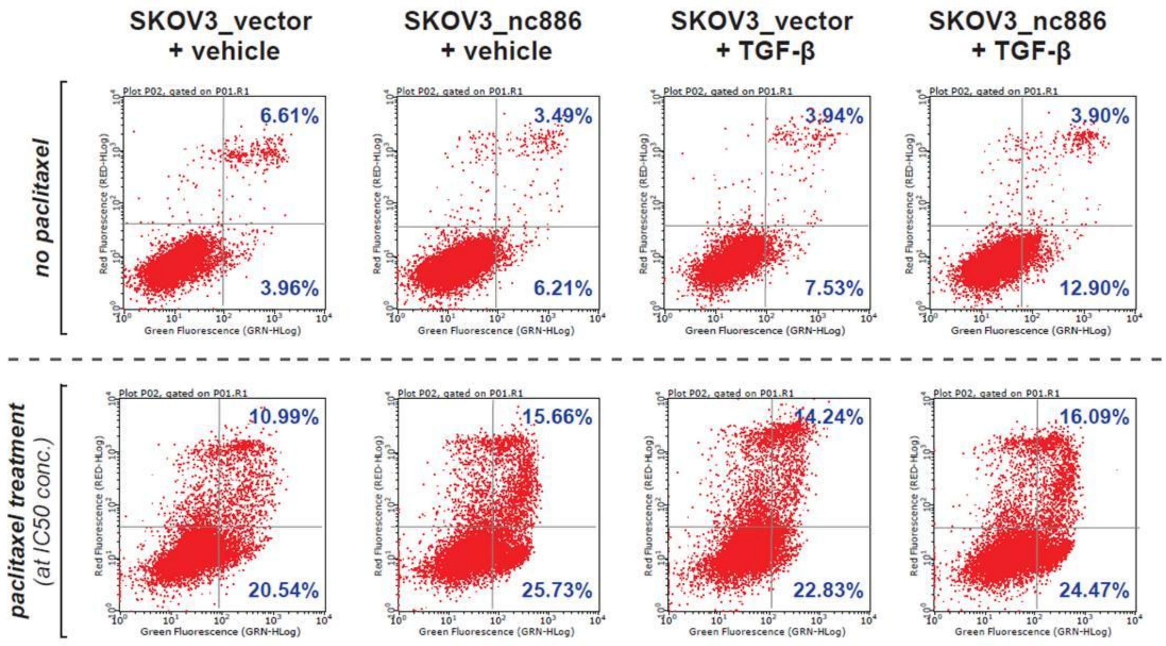

Supplementary Figure 9. Apoptosis assays

SKOV3_vector and SKOV3_nc886 cells were incubated with TGF-β or vehicle for 96 hrs, and then treated with paclitaxel (or vehicle) for 48 hrs by each of the half maximal inhibitory concentration for cell viability (IC50), followed by double staining with PI and FITC-conjugated annexin V. The annexin V-positive cells (apoptotic cells) were detected in the right quadrants of flow cytometry graphs.

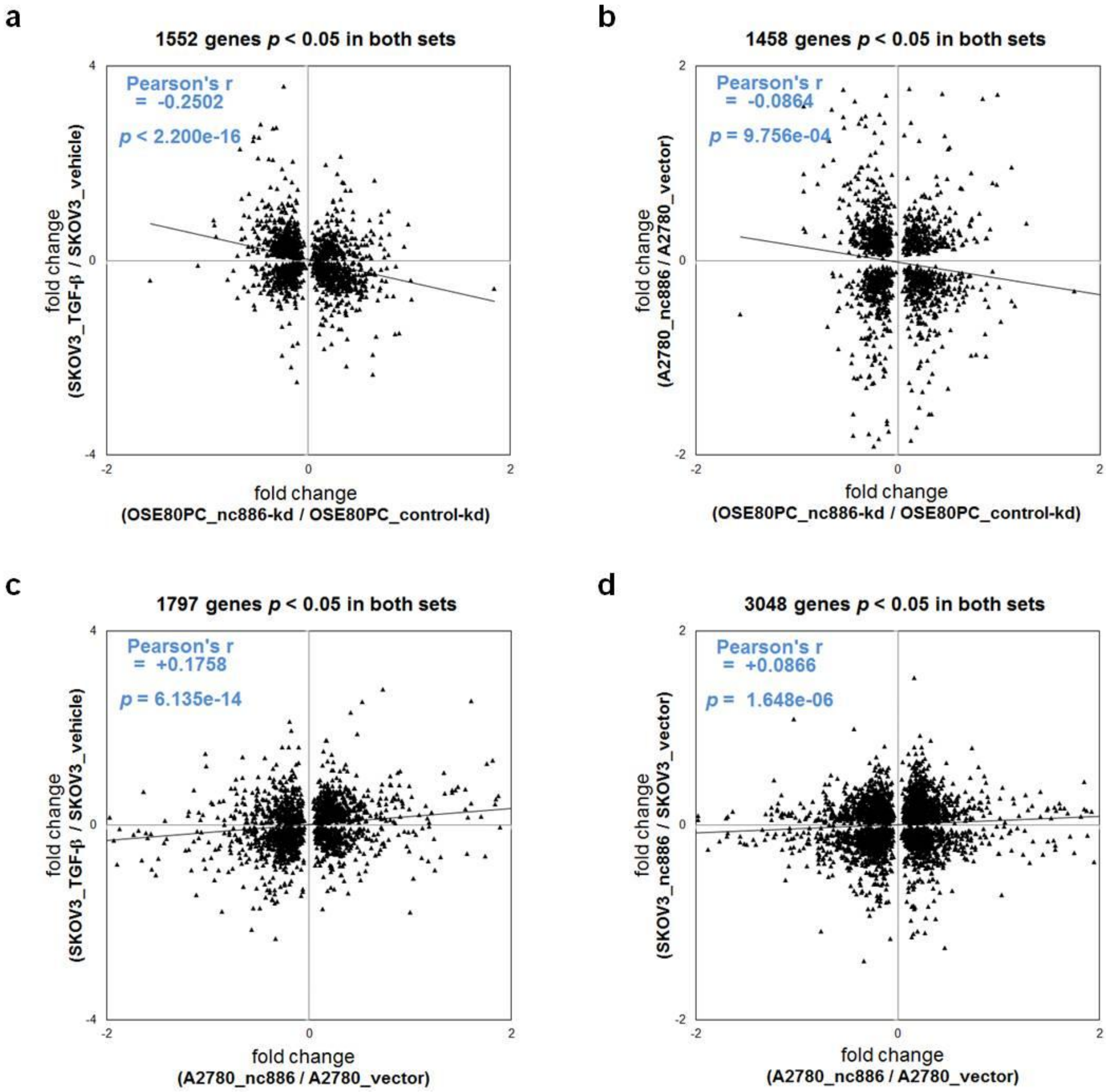

Supplementary Figure 10. Correlation in gene expression; pairwise comparisons among nc886 knockdown (kd), nc886 expression, and TGF-β treatment

Scatter plots of fc values of genes that were significantly changed. In a given pair, genes with *p*-value < 0.05 in both samples were selected. The number of genes is indicated on the top.

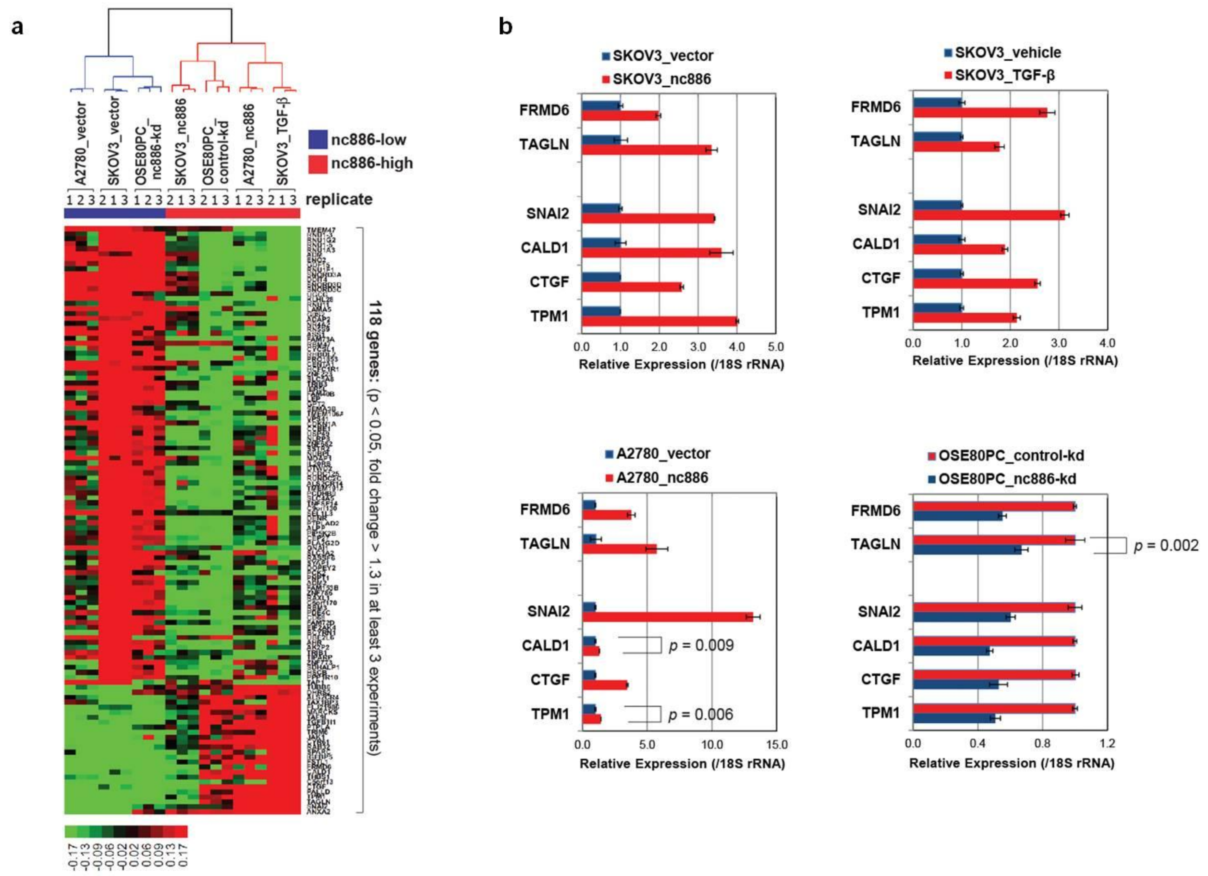

Supplementary Figure 11. 118 genes whose expression level is associated with nc886 in the seven experimental sets

a. A heat map of 118 genes (for a complete list with their array values, see Supplementary Data File 2). The gene selection criterion is described on the right. Their array values relative to the median value across samples are displayed as green to red. The color scale is shown below. As in Fig. 3e, 7 experiments are well partitioned into 2 groups: nc886-low and nc886-high (respectively blue bar and red bar on the top) which are consistent with nc886 levels expected from experimental manipulations (nc886 kd and ectopic expression as well as TGF-β treatment).

b. qRT-PCR of 6 selected genes; 2 (*FRMD6*, *TAGLN*) from the most increased genes in our TGF-β treatment and 4 (*SNAI2*, *CALD1*, *CTGF*, *TPM1*) from TGF-β-induced genes in other studies [5, 6]. qRT-PCR data were consistent with our array data; their expression was induced by TGF-β or nc886, but diminished by nc886 kd. All descriptions are the same as in Supplementary Fig. 1c.

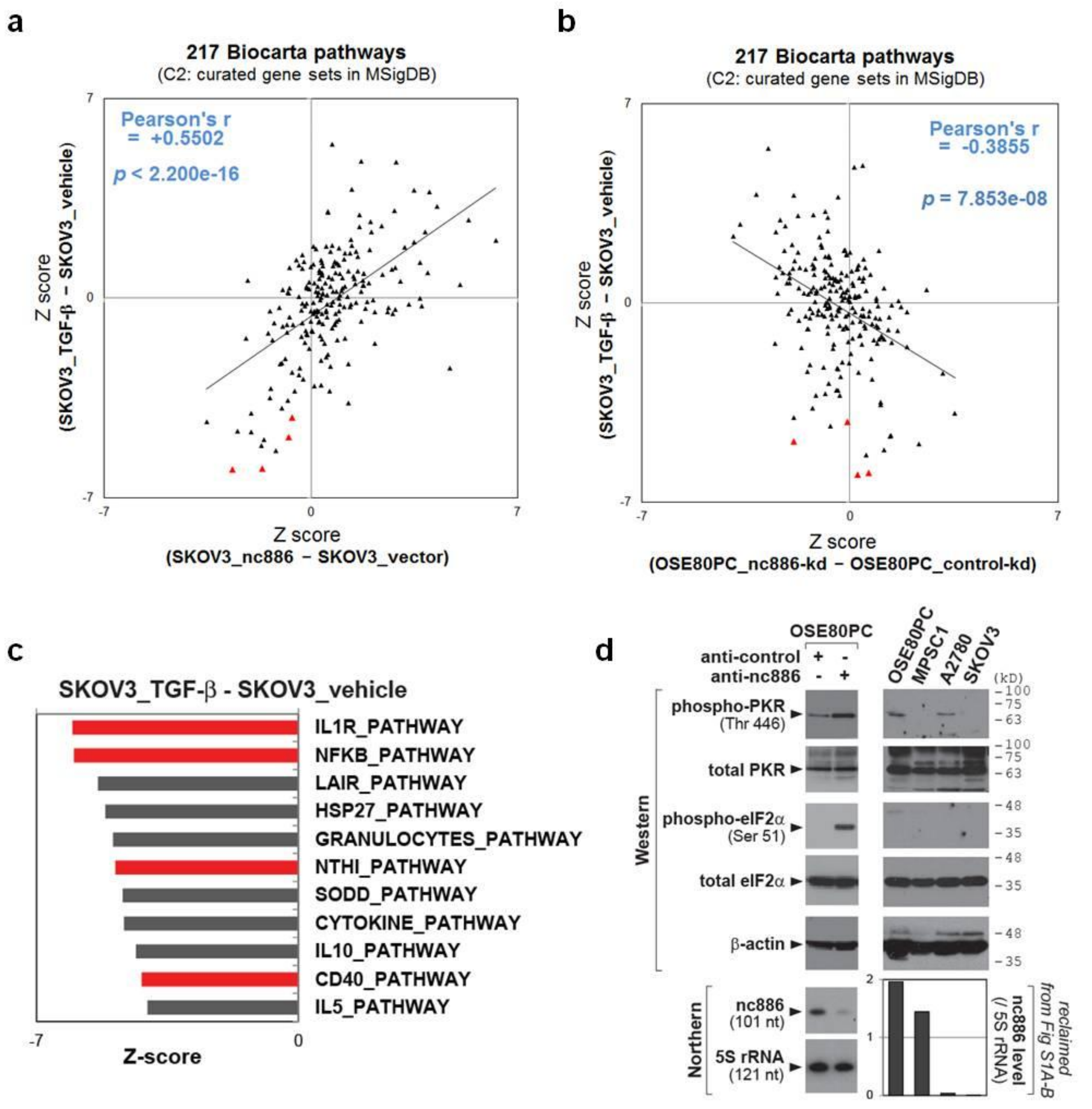

Supplementary Figure 12. nc886-mediated gene regulation cannot be explained by the PKR–NF-κB pathway

We examined the NF-κB and relevant pathways with special attention, because nc886 inhibits PKR and its downstream NF-κB [7] and TGF-β is known to suppress the NF-κB pathway in bacterial infection and inflammation [6, 8]. Actually in our analysis, the NF-κB and relevant pathways (4 red data points in panel a-b and red bars in panel c) were ranked as the most suppressed pathways in TGF-β-treated SKOV3 cells. So, a plausible mechanism is that nc886, which was induced by TGF-β, suppressed the activity of PKR and resultantly also NF-κB. In our analysis, however, nc886 did not elicit significant impact on the NF-κB and relevant pathways. None of these 4 pathways displayed significant Z-scores (>+3 or <-3 to be considered significant) upon nc886 kd or expression (see the location of red data points along x-axis in panel a and b; see also Supplementary Data File 3). In the case of

“OSE80PC_nc886-kd”, PKR was actually activated as indicated by the appearance of phospho-PKR (the active form of PKR) and the resultant phosphorylation of its substrate eIF2α (the left side of panel d). However, this did not result in the activation of NF-κB, suggesting that the PKR–NF-κB connection may have gone awry in OSE80PC cells. In addition, PKR expression data in OC lines also argued against a nc886–PKR–NF-κB relationship. The phospho-PKR bands of OC lines (where nc886 was decreased or silenced) were no more intense than the basal expression in OSE80PC cells (where nc886 expression was high). Likewise, the phospho-eIF2α band was not seen in any of the samples (the right side of panel d). All these data indicated that PKR activation was blocked by a cellular PKR inhibitor other than nc886 in naturally growing OC cells and suggested that nc886’s role in OC metastasis could not be attributed to PKR activation.
a-b. A scatter plot of Biocarta Z-scores between indicated samples. The red data points are NF-κB related pathways (see 4 red bars in panel c).
c. Top suppressed (Z-score cutoff = -4) Biocarta pathways upon TGF-β treatment. NF-κB related pathways are red-highlighted
d. Western blot (top panel) in nc886-kd (48 hrs post-transfection with 100 nM of anti-oligo) and in normally growing ovarian cells. Molecular sizes in kiloDalton (kD) from the size marker are indicated on the right. The information about antibodies is; phospho-PKR (cat# ab32036 from AbCam, Cambridge, MA; diluted to 1/3000), total PKR (cat# sc-707 from Santa Cruz Biotechnology, Dallas, TX; diluted to 1/3000), phospho-eIF2α (cat# 9721S from Cell Signaling Technology, Danvers, MA; diluted to 1/3000), total eIF2α (cat# 9722S from Cell Signaling Technology; diluted to 1/3000), and β-actin (cat# sc-81178 from Santa Cruz Biotechnology; diluted to 1/5000). For nc886 levels, a Northern blot image (bottom left for nc886 kd) and a quantification graph (from Supplementary Fig. 1a-b; bottom right OC lines) are shown.

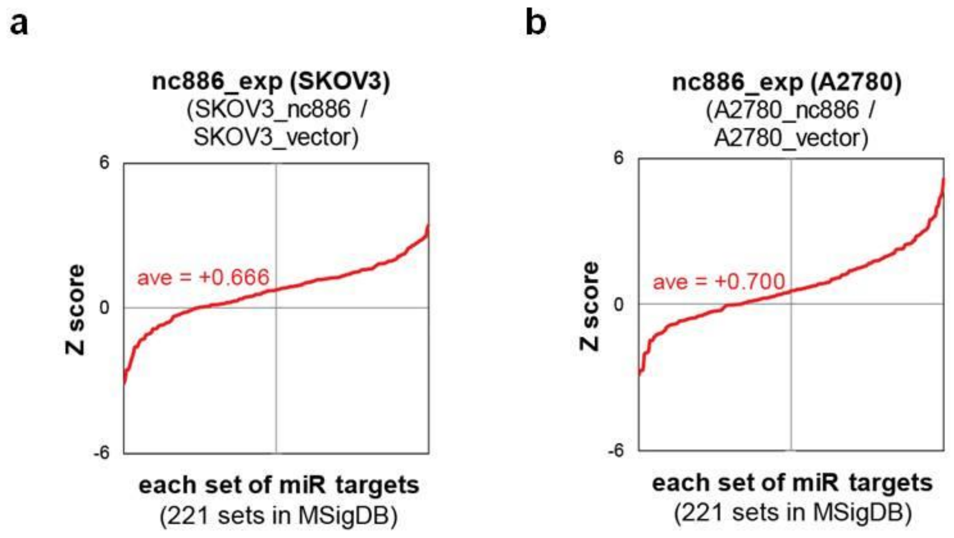

Supplementary Figure 13. MIR Z-scores of nc886-expressing cell lines are inclined toward positive values but less than those of TGF-β treatment

a-b. Rank distribution plots of MIRs in SKOV3 (panel a) and A2780 (panel b) that express nc886 stably. Z-scores were sorted from the smallest to the largest values and plotted against an anonymous x-axis.

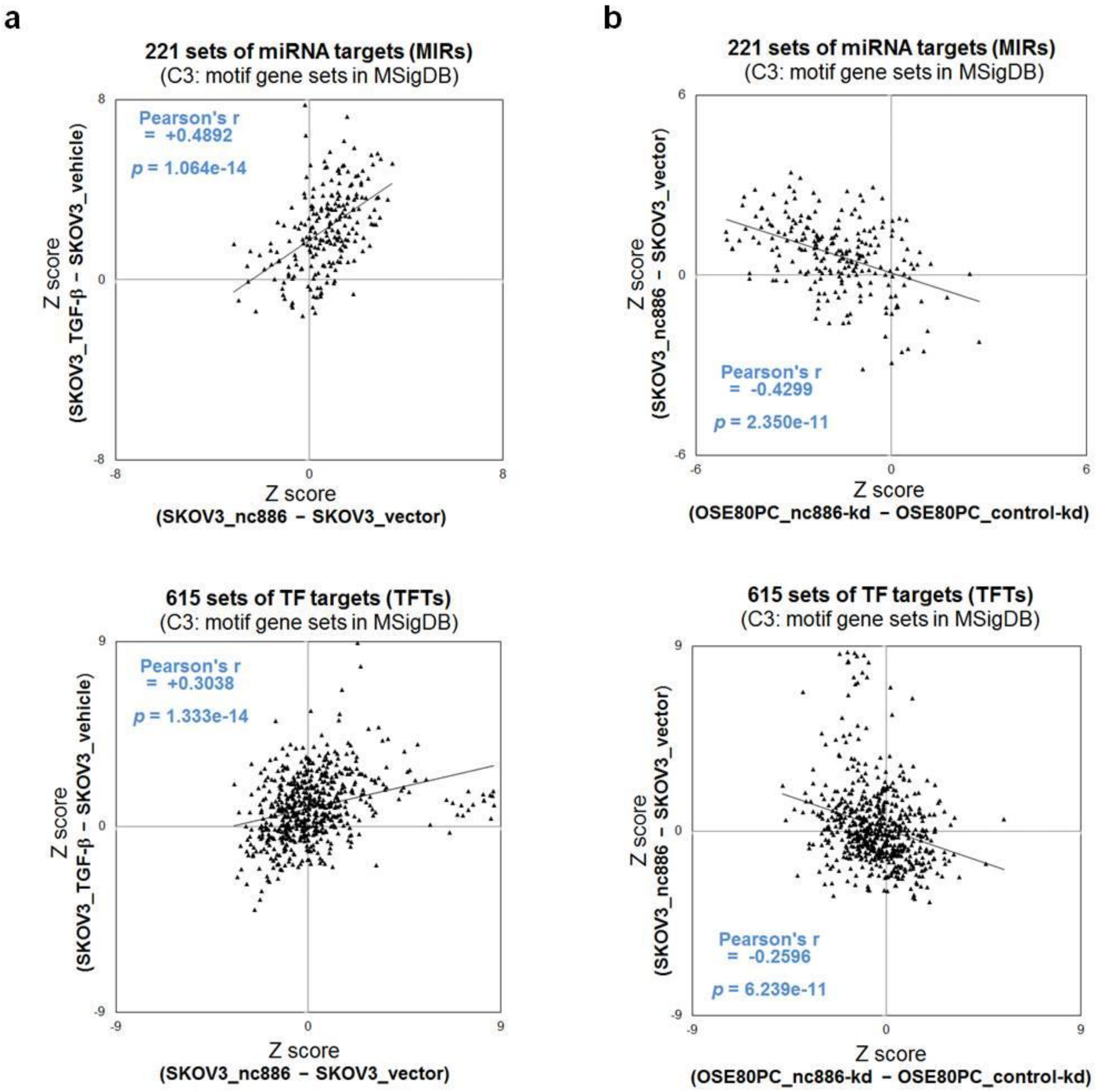

Supplementary Figure 14. MIRs are more correlated with nc886, than with TFTs
a-b. Scatter plots of Z-scores of MIRs (top) and TFTs (bottom) between indicated pairs of datasets.

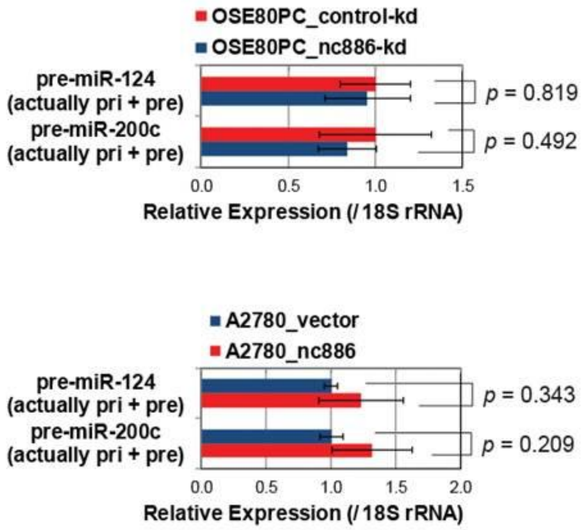

Supplementary Figure 15. qRT-PCR measurement of pre-miRNAs.
qRT-PCR assays to detect pre-miRNAs. The expression levels of these precursors are not significantly altered upon kd or ectopic expression of nc886. Although Dicer inhibition by nc886 led us to expect that pre-miRNAs are consumed and accumulated respectively by nc886-kd and expression, we could not see a statistically significant difference. One reason is that the primers detecting pre-miRNAs inevitably measure also primary miRNA transcripts (pri-miRNAs), so the qRT-PCR signal is not purely from pre-miRNAs. Another reason is that the pre-miRNAs are the processing intermediate and present at a low level as indicated by the high Ct values in our qRT-PCR. Nonetheless, we could exclude a possibility that nc886 suppresses transcription of these miRNAs. If that were the case, pre-miRNA levels (actually a sum of pre- and pri-miRNA levels in our qRT-PCR readout) would have increased upon nc886-kd and decreased in nc886-expressing cells.

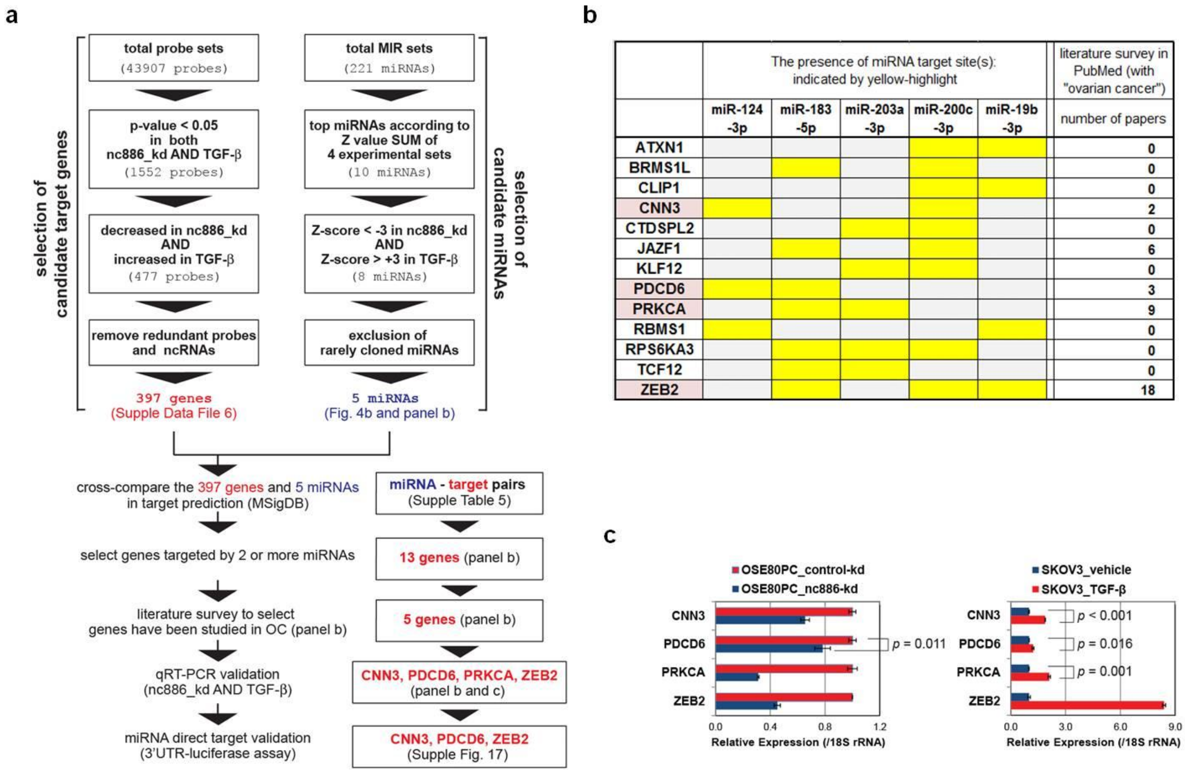

Supplementary Figure 16. Identification of nc886-associated miRNAs and their target genes

a. A workflow to identify miRNAs and target genes relevant to the TGF-β–nc886 pathway in OC. In making shortlists for miRNAs and target mRNAs, greater weight was given to array data from "nc886_kd" and "TGF-β" (short-term treatments within 4 days) than "nc886_exp" (see abbreviations in Fig. 3 for these 3 experimental manipulations), because nc886's direct effect on the miRNA pathway would have diminished in nc886-expressing stable cell lines by secondary effects during their long-term establishment. For candidate miRNA targets, we initially considered 1552 array probes that were altered significantly ($p < 0.05$) in both nc886-kd and TGF-β treatment, and then selected 477 probes that were decreased in nc886-kd, but increased in TGF-β (see a heat map in Fig. 4b and Supplementary Data File 6 for the full list). Elimination of probes for ncRNAs and unannotated genes as well as redundant probes resulted in 397 mRNA genes that were miRNA target candidates. We cast a broad net without applying a fc cutoff because the regulatory capability of a single miRNA on an individual target mRNA is not robust [9, 10]. In selecting miRNAs of higher significance, we considered MIR Z-scores, their abundance estimated from cloning frequencies in the miRNA database (miRbase: www.mirbase.org/). As described in the scheme and the text, we selected 5 miRNAs (miR-124-3p, -183-5p, -203a-3p, -200c-3p, and -19b-3p; see Fig. 4b) for further investigation. We found predicted target genes to be significantly enriched in the 397 genes when the MIR gene sets for these 5 miRNAs were analyzed. For example, 552 genes are listed in the MIR set of miR-124-3p (designated as miR-124a in the MSigDB), which calculates to be ~2.8% of the 19,313 protein-coding genes in the human genome. When we analyzed the 397 genes against this MIR set, 25 genes (6.3%) were found to be targeted by

miR-124-3p (enrichment *p*-value < 0.001). This enrichment was seen in all the other 4 miRNAs (see Supplementary Table 5), supporting our view that nc886-kd and TGF-β modulates the miRNA pathway to influence the gene expression pattern.

b. 13 candidate miRNA target genes harboring recognition sites for 2 or more miRNAs (yellow-highlighted boxes). When making a shorter list for candidate target genes, we chose genes that were targeted by more than one miRNA, because multiple miRNAs restraining a single gene would obviously be more suppressive than a single miRNA, via cooperative or simply additive action [10]. Pink highlighted genes are ones that were implicated in OC in previous studies (surveyed in the PubMed database: http://www.ncbi.nlm.nih.gov/pubmed) and that were validated by qRT-PCR in panel c.

c. qRT-PCR of the 4 genes, all of which were validated to be decreased upon nc886 kd but increased upon TGF-β treatment.

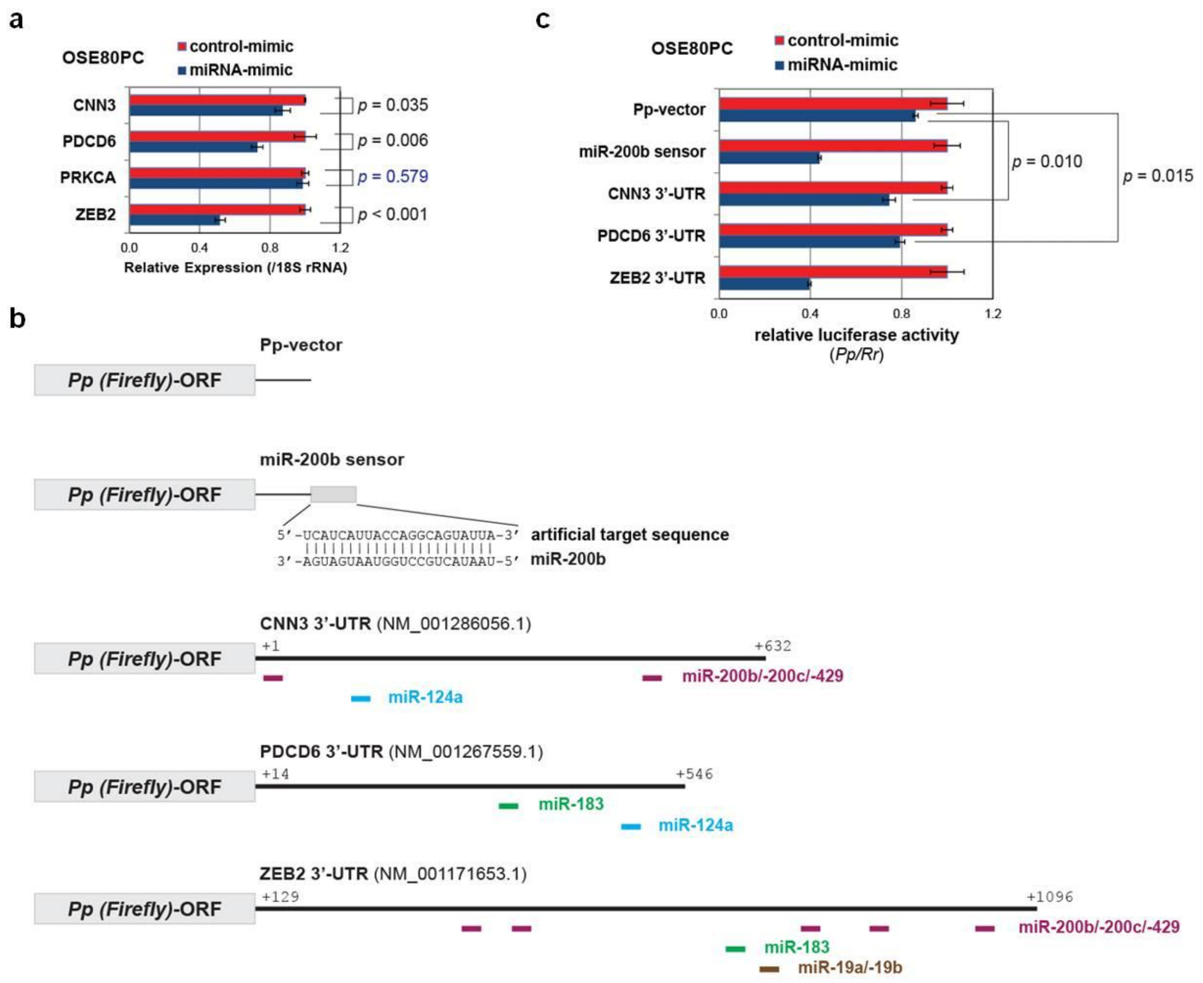

Supplementary Figure 17. Validation of direct miRNA targets

a. qRT-PCR of the 4 genes upon transfection of miRNA-mimic (a mixture of miR-124-3p, -183-5p, -203a-3p, -200c-3p, and -19b-3p) or a non-targeting control-mimic was transfected into OSE80PC cells, followed by cell harvest for RNA preparation after 24 hrs. mRNA expression of 3 genes (*CNN3*, *PDCD6*, and *ZEB2*), but not of *PRKCA*, was repressed by miRNA mimic, indicating that *PRKCA* was not regulated by these miRNAs. Therefore, we proceeded with the 3 genes for validation by luciferase reporter assays (panel b-c).

b. A diagram showing 3'-untranslated regions of candidate miRNA target genes. These regions were cloned into a plasmid at the downstream of firefly luciferase (*Pp*) open reading frame. As a positive control, an artificially designed perfect complementary target site was also cloned. Detailed information on the plasmids is described in Supplementary Table 4 (and also Supplementary Data File 9 for primers used to construct these plasmids).

c. Luciferase assays upon transfection of the miRNA-mimic (the same mixture as in panel a) or the control-mimic, together with the plasmids in panel b. Assays were performed at 24 hrs after transfection of luciferase plasmids. The relative luciferase values (y-axis) were calculated through a couple of normalizations. Initially, values from *Pp* were normalized to Renilla luciferase (*Rr*) values from co-transfected pRL-SV40. Then, in each plasmid, the *Pp*/*Rr* value of the negative control mimic was set as 1. An average and a standard deviation

were calculated from triplicate samples. In all of the 3 genes, their 3'-untranslated regions harboring miRNA target sites were sufficient to confer repression by the miRNA mimic in luciferase assays, demonstrating that *CNN3*, *PDCD6*, and *ZEB2* were direct miRNA targets in the TGF-β–nc886 pathway.

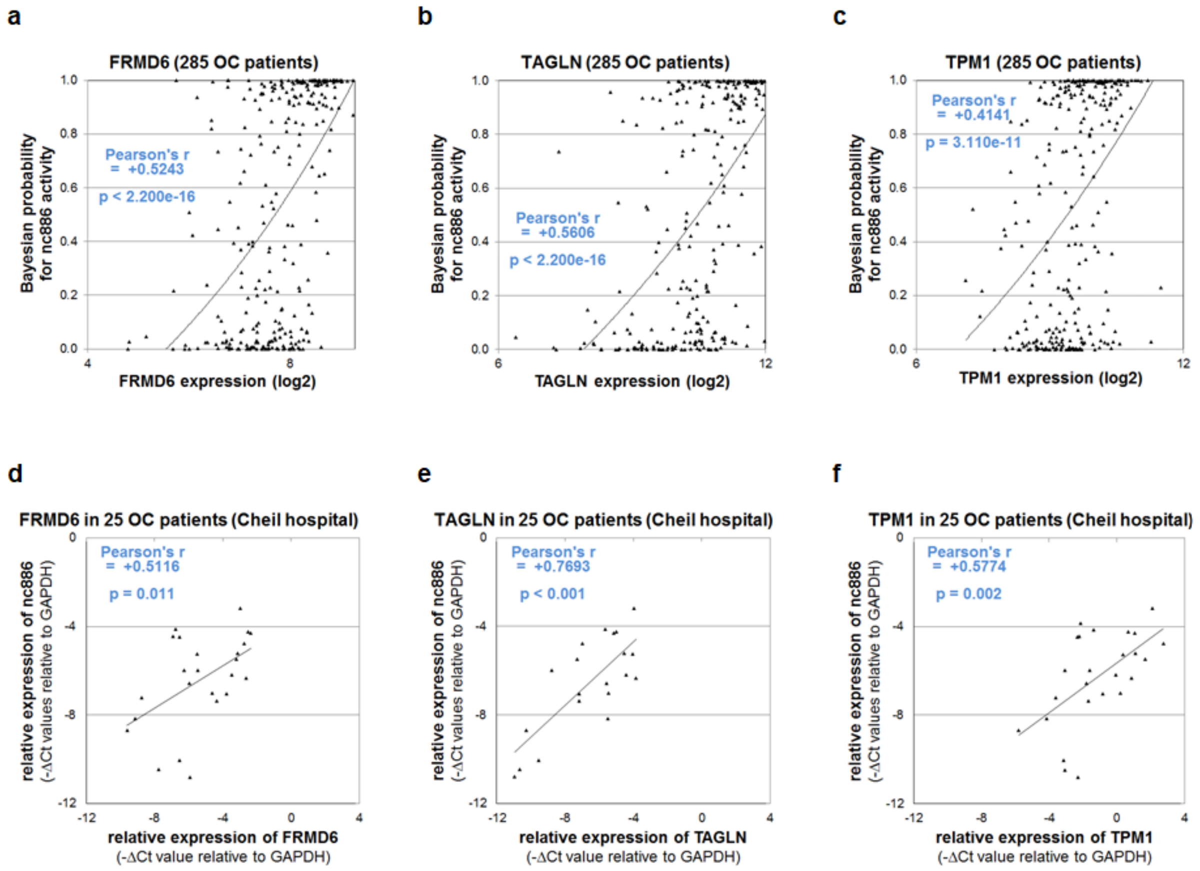

Supplementary Figure 18. Validation of proxy genes by comparing to nc886

a-c. From the 118 gene signature (shown in Supplementary Fig. 11a, listed in Supplementary Data File 2, and used for clinical analysis in Fig. 7), 3 genes (*FRMD6*, *TAGLN*, and *TPM1*) were selected for the following reasons. First, they were altered in an anticipated direction (decreased upon "nc886_kd" and increased upon "nc886_exp" or "TGF-β") in our model that nc886 inhibits the miRNA pathway and therefore increases miRNA target genes. Second, they were among the top regulated genes by nc886 and TGF-β (ranked 8, 9, 17th when we summated the fold-change in the 4 experimental pairs). Third, their expression was confirmed by qRT-PCR (Supplementary Fig. 11b). In the cohort of OC patients (GSE9891, n=285) that was analyzed for nc886's clinical significance in Fig. 7, the expression level of each of the 3 genes (normalized microarray values on x-axis) was plotted against the 118-gene signature (expressed in BCCP probability values on y-axis). There was a significant positive correlation, indicating that *FRMD6*, *TAGLN*, and *TPM1* are good representative genes of the 118-gene signature.

d-f. The 3 genes (*FRMD6*, *TAGLN*, and *TPM1*) and nc886 were measured by qRT-PCR in patients from the Cheil General Hospital and Women's Healthcare Center (abbreviated as "Cheil cohort" in the main text). –ΔCt values of each of the 3 genes are plotted against those of nc886. All the 3 genes display significant correlation to nc886. Taken together with our data in panel a-c, these qRT-PCR data corroborated that the 118-gene signature can be used as a proxy marker for the expression of nc886.

Supplementary Table 1. Summary of ovarian cell lines in this study

name	characteristics	source	cell check	growth media	nc886 level
**primary OSE**	primary ovarian surface epithelial cells	ScienCell Research Laboratories (cat # 7310)	from bank	OEpiCM	++
**IOSE-80PC (OSE80PC)**	immortalized ovarian surface epithelial cells	Dr. Nelly Auersperg (University of British Columbia, Vancouver, British Columbia, Canada)	yes	RPMI	+++++
**MPSC1 (JH514)**	low grade ovarian serous adenocarcinoma	Dr. Ie-Ming Shih (John Hopkins Medical Institutions, Baltimore, MD, USA)	yes	RPMI	+++
**HeyA8**	high grade ovarian adenocarcinoma	Dr. Anil K. Sood (MD Anderson Cancer Center, Houston, TX, USA)	yes	RPMI	+++++++
**OVCA5**	high grade ovarian adenocarcinoma	Dr. Anil K. Sood (MD Anderson Cancer Center, Houston, TX, USA)	yes	RPMI	+++++++++
**OVCA420**	ovarian adenocarcinoma	Dr. Anil K. Sood (MD Anderson Cancer Center, Houston, TX, USA)	yes	RPMI	+
**OVCAR-3**	high grade ovarian adenocarcinoma	Dr. Ie-Ming Shih (John Hopkins Medical Institutions, Baltimore, MD, USA)	yes	RPMI	+++
**A2780**	ovarian adenocarcinoma	Dr. Ie-Ming Shih (John Hopkins Medical Institutions, Baltimore, MD, USA)	yes	RPMI	-
**SKOV3**	ovarian adenocarcinoma	Dr. Ie-Ming Shih (John Hopkins Medical Institutions, Baltimore, MD, USA)	yes	RPMI	-
**OVCA433**	ovarian adenocarcinoma	Dr. Anil K. Sood (MD Anderson Cancer Center, Houston, TX, USA)	yes	RPMI	-
**OVCA432**	ovarian adenocarcinoma	Dr. Anil K. Sood (MD Anderson Cancer Center, Houston, TX, USA)	yes	RPMI	-
**IGROV-1**	ovarian adenocarcinoma	Dr. Anil K. Sood (MD Anderson Cancer Center, Houston, TX, USA)	yes	RPMI	-

media information		
**OEpiCM**	Ovarian Epithelial Cell Medium	cat # 7311 from ScienCell Research Laboratories (reconstituted as instructed by the manufacturer)
**RPMI**	RPMI Medium 1640	cat # 11875-093 from ThermoFisher Scientific (supplemented with 10% FBS, 1% antibiotics)
**FBS**	Fetal Bovine Serum	cat # S11550 from Atlanta Biologicals
**antibiotics**	Penicillin-Streptomycin	cat # 15140122 from ThermoFisher Scientific

Supplementary Table 2. Methylation values of CpG sites in EpiTYPER assays (raw values for Fig. 1g)

	SKOV3		
**CpG sites***	**untreat**	**TGF-β**	**AzadC**
-127 & -120	0.89	0.57	0.52
-113 & -109	0.91	0.58	0.53
-85 & -79	0.95	0.57	0.50
-61	0.91	0.64	0.59
-46	1.00	0.74	0.61
-15	0.76	0.47	0.38
-1 & +5	0.88	0.52	0.42
+72	0.94	0.69	0.64
+79 & +81	0.92	0.69	0.61
+253 & +257	0.96	0.64	0.69
+359 & +361	0.99	0.55	0.73
+368	0.96	0.69	0.74
+380	0.96	0.71	0.68
+418	0.94	0.62	0.57
+467	0.97	0.43	0.51

* nucleotide coordinates relative to the 5'-end of the nc886 transcript as +1

Supplementary Table 3. Stable cell lines constructed and used in this study

cell line designation in Figures and texts (see Supplementary Fig. 5a and 5c)	parental line	plasmid used (see Supplementary Table 4)	Selection marker information	comments
SKOV3_vector	SKOV3	pLKO.1 - TRC	puromycin* 200 ng/ml	mostly used in main Figures (functional assays and mRNA array)
SKOV3_nc886	SKOV3	pLKO.1-886(102T)		
A2780_vector	A2780	pCAGGS-GFP	G418** 500 ng/ml	
A2780_nc886	A2780	pCAGGS-GFP/886		
SKOV3-GFP (vector)	SKOV3	pCAGGS-GFP	G418 500 ng/ml	used in Supplemental Figures (functional assays)
SKOV3-GFP (nc886)	SKOV3	pCAGGS-GFP/886		
A2780-pLKO (vector)	A2780	pLKO.1 - TRC	puromycin 200 ng/ml	
A2780-pLKO (nc886)	A2780	pLKO.1-886(102T)		

puromycin* : cat # A11138-03 from Invitrogen
G418** : cat # MT30005CR from ThermoFisher Scientific

Supplementary Table 4. Plasmid DNAs used in this study

**description in this work**	**plasmid name**	**source**	**backbone vector**	**insert** (see Supplementary Data File 9 for primer information)
				for transient expression of nc886
**vector_control**	pLPCX-U6	Lee laboratory	pLPCX (Clontech)	U6 promoter from pMSCV-U6 (PCR amplified with "U6pro F-BglII" and "U6pro R-EcoRI")
**nc886_expression**	pLPCX-U6-pre-886	Lee laboratory	pLPCX-U6	nc886 (102 bp PCR product with "miR-886-5p sense" and "pre-886 as RI")
				for nc886 stable cell line (see Supplementary Fig. 5a and 5c)
	pCAGGS-GFP	Jeon laboratory	pCAGGS-Neo	GFP
	pCAGGS-GFP/886	Lee laboratory	pCAGGS-GFP	nc886 and its flanking genomic sequence (649 bp PCR product with "miR886 41-60" and "miR886 689-70")
	pLKO.1 - TRC	Addgene		
	pLKO.1-886(102T)	Lee laboratory	pLKO.1 - TRC	nc886 (102 bp PCR product with "nc886 1-18 AgeI" and "pre-886 as RI")
				for DNMT expression
**empty vector**	pcDNA-YFP	a gift from Dr. François Fuks	pcDNA-YFP	
	pcYFP_Dnmt1	a gift from Dr. François Fuks	pcDNA-YFP	
				for Dicer-nc886 interaction
**empty vector**	pcDNA3-FLAG	a gift from Dr. Yong-Soo Bae	pcDNA3.1	
	pcDNA3.1-FLAG-Dicer (WT)	a gift from Dr. Narry Kim	pcDNA3.1	
	pcDNA3.1-FLAG-Dicer (DDUF)	a gift from Dr. Narry Kim	pcDNA3.1	
**wtDicer**	pcDNA3.1-FLAG-Dicer (DPAZ)	a gift from Dr. Narry Kim	pcDNA3.1	
				for luciferase assays for putative miRNA target genes
**Rr-vector**	pRL-SV40	Promega		
**Pp-vector**	pcDNA3-1 Zeo(+)-Pp	Lee laboratory	pcDNA3-1 Zeo(+)	firefly luciferase (Pp) ORF (nt # 251-1932 of pGL3-Control from Promega)
**miR-200b sensor**	pc3miR200b3p-as	Lee laboratory	pcDNA3-1 Zeo(+)-Pp	perfect antisense to miR-200b-3p (by annealing "miR-200b-3p sense" and "miR-200b-3p as X/A")
**CNN3 3'-UTR**	pc3Pp_CNN3UTR	Lee laboratory	pcDNA3-1 Zeo(+)-Pp	3'UTR of CNN3 (632 bp PCR product with "CNN3 1110-32" and "CNN3 1741-19")
**PDCD6 3'-UTR**	pc3Pp_PDCD6UTR	Lee laboratory	pcDNA3-1 Zeo(+)-Pp	3'UTR of PDCD6 (533 bp PCR product with "PDCD6 324-46" and "PDCD6 856-33")
**ZEB2 3'-UTR**	pc3Pp_ZEB2UTR	Lee laboratory	pcDNA3-1 Zeo(+)-Pp	3'UTR of ZEB2 (968 bp PCR product with "ZEB2 4224-46" and "ZEB2 5191-68")

Supplementary Table 5. Candidate target genes of the five miRNAs. Genes targeted by more than one miRNA out of the five nc886 signature miRNAs are bold-highlighted

**miRNAs**	**miR-124-3p**	**miR-183-5p**	**miR-203a-3p**	**miR-200c-3p**	**miR-19b-3p**
**seed sequence**	TGCCTTA	GTGCCAT	CATTTCA	CAGTATT	TTTGCAC
**total # of targets**	552	175	287	469	516
**# of targets in 397 genes**	25	15	15	14	17
**enrichment *p*-value**	0.000005	0.000055	0.001991	0.009299	0.022407
**target genes**	AKT3	AMD1	**CTDSPL2**	**ATXN1**	**ATXN1**
	ARPC5	**BRMS1L**	IGFBP5	BCAP29	**CLIP1**
	B4GALT1	FRMD6	KIF2A	**BRMS1L**	CTGF
	BRP44L	**PDCD6**	**KLF12**	**CLIP1**	FASTK
	CASC4	PHLDB2	LAMP2	**CNN3**	HECW2
	**CNN3**	PPP2CB	MAPK9	**CTDSPL2**	IVNS1ABP
	COL12A1	**PRKCA**	NUAK1	FEZ2	KIT
	COL4A1	RCN2	PHF19	**JAZF1**	MEMO1
	CUL5	**RPS6KA3**	PPP1CB	**KLF12**	MMGT1
	DNAJB12	**TCF12**	**PRKCA**	KRT80	RAB2B
	**JAZF1**	TLE4	**RPS6KA3**	MARCKS	**RBMS1**
	KIAA1024	TMPO	SPARC	**RPS6KA3**	SYT11
	LEMD3	TPM1	**TCF12**	TUBB	TGOLN2
	MAP1B	TRAM1	VCAN	**ZEB2**	TNFRSF12A
	MPZL1	ZMYM2	XRN2		TRAK2
	MYADM				UBL3
	MYH9				**ZEB2**
	PALLD				
	**PDCD6**				
	PLSCR3				
	PRRX1				
	**RBMS1**				
	SLC7A8				
	SNX6				
	TOR3A				

Supplementary Table 6. Clinicopathologic characteristics of 285 ovarian cancer patients

	nc886 high	nc886 low	NA
**Age at diagnosis**			
Median	60.74	57.39	58.47
Range	22.85-79.92	22.36-80.05	47.05-75.99
**Stage**			
I	7	14	3
II	3	13	2
III	109	82	26
IV	15	5	2
Unknown	3	1	0
**Histologic subtype**			
Serous	130	102	32
Endometrioid	7	12	1
Adenocarinoma	0	1	0
**Grade**			
1	5	14	0
2	49	37	11
3	80	62	22
unknown	3	2	0
**Primary site**			
Ovary	107	110	26
Fallopian Tube	3	3	2
Peritoneum	27	2	5
**Residual disease**			
Nil macroscopic	26	48	10
<=1 cm	37	29	11
>1cm	48	23	10
Unknown	26	15	2

Supplementary Table 7. Univariate and multivariate Cox proportional hazard regression analyses for overall survival

	Univariate		Multivariate	
	Hazard Ratio (95% CI)	*P*-value	Hazard Ratio (95% CI)	*P*-value
**Age (>50)**	1.2 (0.69 – 2.1)	0.49	1.3 (0.75 - 2.3)	0.31
**Stage (I/II or III/IV)**	6.4 (2.0 - 20.4)	0.001	2.4 (0.68 – 8.5)	0.16
**Grade (1 or 2/3)**	2.8 (0.89 - 8.9)	0.07	1.7 (0.38 - 7.2)	0.48
**Residual disease (absence or presence)**	3.7 (2.0 - 6.9)	> 0.001	2.3 (1.18 – 4.4)	0.01
**nc886 (low or high)**	2.0 (1.33 - 3.1)	0.001	1.7 (1.1 - 2.8)	0.01

Supplementary References

1. Lee YS, Dutta A. The tumor suppressor microRNA let-7 represses the HMGA2 oncogene. *Genes Dev* **21**, 1025-1030 (2007).

2. Jung SY, *et al.* An Anatomically Resolved Mouse Brain Proteome Reveals Parkinson Disease-relevant Pathways. *Mol Cell Proteomics* **16**, 581-593 (2017).

3. Park JL, *et al.* Epigenetic regulation of RNA polymerase III transcription in early breast tumorigenesis. *Oncogene*, (2017).

4. Kennedy BA, *et al.* ChIP-seq defined genome-wide map of TGFbeta/SMAD4 targets: implications with clinical outcome of ovarian cancer. *PLoS One* **6**, e22606 (2011).

5. Cheon DJ, *et al.* A collagen-remodeling gene signature regulated by TGF-beta signaling is associated with metastasis and poor survival in serous ovarian cancer. *Clin Cancer Res* **20**, 711-723 (2014).

6. Yeung TL, *et al.* TGF-beta modulates ovarian cancer invasion by upregulating CAF-derived versican in the tumor microenvironment. *Cancer Res* **73**, 5016-5028 (2013).

7. Marchal JA, *et al.* The impact of PKR activation: from neurodegeneration to cancer. *FASEB J* **28**, 1965-1974 (2014).

8. Guo X, Wang XF. Signaling cross-talk between TGF-beta/BMP and other pathways. *Cell Res* **19**, 71-88 (2009).

9. Lee YS, Dutta A. MicroRNAs in cancer. *Annu Rev Pathol* **4**, 199-227 (2009).

10. Bartel DP. MicroRNAs: target recognition and regulatory functions. *Cell* **136**, 215-233 (2009).
