# Supplementary material for: nc886 is induced by TGF-β and suppresses the microRNA pathway in ovarian cancer
Source: Nat Commun. 2018 Mar 21;9:1166. doi: 10.1038/s41467-018-03556-7 (PMC5862949; doi:10.1038/s41467-018-03556-7)
Supplement: Supplementary file 3 — Description of Additional Supplementary Files [file 41467_2018_3556_MOESM3_ESM.pdf]

Description of Additional Supplementary Files

File Name: Supplementary Data File 1
Description: Fold changes and *p*-values of TGF-β- and nc886-induced genes (in Fig. 3a)

File Name: Supplementary Data File 2
Description: Array values (relative to the median value) of 118 nc886-associated genes in the 7 experimental sets (in Supplementary Fig. 11a)

File Name: Supplementary Data File 3
Description:Z-scores of 217 sets of Biocarta pathways ("BIOCARTA" from C2: curated gene sets in MSigDB).

File Name: Supplementary Data File 4
Description: Z-scores of 221 sets of miRNA targets ("MIR" from C3: motif gene sets in MSigDB)

File Name: Supplementary Data File 5
Description: Z-scores of 615 sets of TF targets ("TFT" from C3: motif gene sets in MSigDB)

File Name: Supplementary Data File 6
Description: Fold changes and *p*-values of 477 probes (containing 397 candidate miRNA target genes) selected from nc886 knockdown and TGF-β treatment (Fig. 4b)

File Name: Supplementary Data File 7
Description: nc886-specific interacting proteins found in mass spectrometry analysis. Known DICER1 direct binding proteins from STRING™ (shown in Fig. 5a) are highlighted in bold.

File Name: Supplementary Data File 8
Description: Expression of the three genes (*FRMD6*, *TAGLN*, and *TPM1*) and the Bayesian probability score of the 118-gene nc886 signature in the OC patient cohort (GSE9891, n=285)

File Name: Supplementary Data File 9
Description: Primers used in this study
